# Dibenzoato-κ*O*;κ^2^ *O*,*O*′-(6,6′-dimethyl-2,2′-bipyridine-κ^2^ *N*,*N*′)zinc(II)–benzoic acid (1/1)

**DOI:** 10.1107/S1600536809042093

**Published:** 2009-10-23

**Authors:** Li Yao, Wen-Juan Li

**Affiliations:** a School of Computer and Information Engineering, Henan University, Kaifeng 475001, Henan, People’s Republic of China; b Department of Civil and Environmental Engineering, East China Institute of Technology, 56 Xuefu Road, Fuzhou 344000, Jiangxi, People’s Republic of China

## Abstract

In the crystal structure of the title compound, [Zn(C_6_H_5_COO)_2_(C_12_H_12_N_2_)]·C_6_H_5_COOH, the Zn atom is penta­coordinated in distorted square-pyramidal geometry by two O atoms of a benzoate anion and two N atoms of a 6,6′-dimethyl-2,2′-bipyridine ligand occupying the basal plane and an O atom of another benzoate anion located at the apical site. In the crystal structure, inter­molecular O—H⋯O and C—H⋯O hydrogen bonds and C—H⋯π inter­actions are present.

## Related literature

For related structures, see: Alizadeh *et al.* (2009 ▶); Cui *et al.* (2005 ▶); Hökelek *et al.* (2009*a* ▶,*b* ▶); Klausmeyer *et al.* (2007 ▶); Phatchimkun *et al.* (2009 ▶); Zhang *et al.* (2009 ▶).
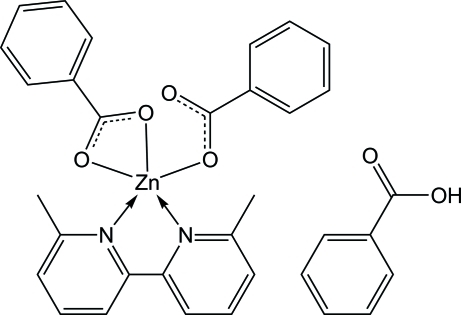


## Experimental

### 

#### Crystal data

[Zn(C_7_H_5_O_2_)_2_(C_12_H_12_N_2_)]·C_7_H_6_O_2_
*M* *_r_* = 613.96
Triclinic, 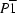

*a* = 9.974 (6) Å
*b* = 12.546 (7) Å
*c* = 12.798 (8) Å
α = 96.631 (11)°
β = 97.016 (12)°
γ = 105.060 (12)°
*V* = 1516.7 (16) Å^3^
*Z* = 2
Mo *K*α radiation
μ = 0.86 mm^−1^
*T* = 296 K
0.16 × 0.14 × 0.12 mm

#### Data collection

Bruker APEXII CCD area-detector diffractometer
Absorption correction: multi-scan (*SADABS*; Bruker, 2005 ▶) *T*_min_ = 0.875, *T*_max_ = 0.904
8123 measured reflections
5295 independent reflections
2785 reflections with *I* > 2σ(*I*)
*R*_int_ = 0.046

#### Refinement

*R*[*F* ^2^ > 2σ(*F* ^2^)] = 0.065
*wR*(*F* ^2^) = 0.136
*S* = 1.04
5295 reflections
370 parameters
14 restraints
H-atom parameters constrained
Δρ_max_ = 0.64 e Å^−3^
Δρ_min_ = −0.64 e Å^−3^

### 

Data collection: *APEX2* (Bruker, 2005 ▶); cell refinement: *SAINT* (Bruker, 2005 ▶); data reduction: *SAINT*; program(s) used to solve structure: *SHELXS97* (Sheldrick, 2008 ▶); program(s) used to refine structure: *SHELXL97* (Sheldrick, 2008 ▶); molecular graphics: *SHELXTL* (Sheldrick, 2008 ▶); software used to prepare material for publication: *SHELXTL*.

## Supplementary Material

Crystal structure: contains datablocks I, global. DOI: 10.1107/S1600536809042093/rk2173sup1.cif

Structure factors: contains datablocks I. DOI: 10.1107/S1600536809042093/rk2173Isup2.hkl

Additional supplementary materials:  crystallographic information; 3D view; checkCIF report

## Figures and Tables

**Table 1:** Hydrogen-bond geometry (Å, °)

*D*—H⋯*A*	*D*—H	H⋯*A*	*D*⋯*A*	*D*—H⋯*A*
C24—H24⋯O5^i^	0.93	2.44	3.340 (8)	162
C33—H33*C*⋯O1	0.96	2.52	3.199 (8)	128
C22—H22*A*⋯O2	0.96	2.57	3.289 (7)	132
O6—H6⋯O4^ii^	0.82	1.85	2.655 (5)	166
C31—H31⋯*Cg*1^iii^	0.93	2.79	3.708 (7)	170

Symmetry codes: (i) 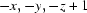; (ii) 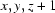; (iii) 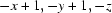. *Cg*1 is the centroid of the C9–C14 ring.

**Fig. 1.**
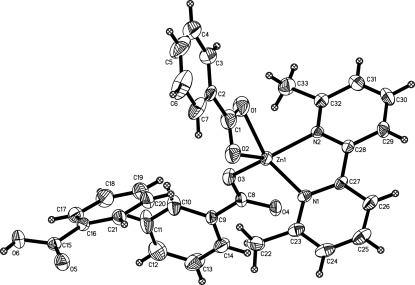
The molecular structure of the title compound with the atom-labelling scheme. Displacement ellipsoids are drawn at the 30% probability level. H atoms are presented as small spheres of arbitrary radius.

## supplementary crystallographic information

### Comment

As a contribution to structural characterization of 6,6'-dimethyl-2,2'-bipyridine complexes [Alizadeh *et al.*, (2009); Cui *et al.*, (2005); Hökelek *et al.*, (2009a,b); Klausmeyer *et al.*, (2007); Phatchimkun *et al.* (2009); Zhang *et al.*, (2009)], we present here the molecular structure of the title complex, Zn*L*(C_6_H_5_COO)_2_^.^C_6_H_5_COOH, where *L* is 6,6'-dimethyl-2,2'-bipyridine.

The title compound, contains two benzoate anions, one 6,6'-dimethyl-2,2'-bipyridine ligand and one benzoic acid molecules. One of the benzoate anions acts as a bidentate ligand, while the other is monodentate.

The molecular structure of the title complex is shown on Fig. 1. In the crystal structure, the face-to-face separation of 3.783 (4)Å suggests no π···π stacking between parallel bipyridine ring systems, intermolecular O—H···O and C—H···O hydrogen bonds (Table 1) link the molecules into a one dimensional structure, in which they may be effective in the stabilization of the structure. One weak C—H···π interactions (Table 1) are also found. *Cg*1 is the centroid of the C9^iii^-C14^iii^ ring. Symmetry code: (iii) 1-*x*, 1-*y*, -*z*.

### Experimental

The title complound was synthesized hydrothermally in a teflon-lined autoclave (25 ml) by heating a mixture of 6,6'-dimethyl-2,2'-bipyridine (0.2 mmol), benzoic acid (0.4 mmol) and ZnSO_4_^.^H_2_O (0.2 mmol) in water (10 ml) at 393 K for 3 d. Crystals suitable for X-ray analysis were obtained.

### Refinement

The carboxy H atom was located in a difference Fourier map and refined with a O—H distance of 0.82Å [*U*_iso_(H) = 1.5*U*_eq_(O)]. H atoms bonded to C atoms were positioned geometrically and refined using a riding model with C—H = 0.93Å and 0.96Å for aromatic and methyl H atoms, respectively, and *U*_iso_(H) values were calculated at 1.5*U*_eq_(C) for methyl groups and 1.2*U*_eq_(C) for aromatic.

### Crystal data

**Table:**

[Zn(C_7_H_5_O_2_)_2_(C_12_H_12_N_2_)]·C_7_H_6_O_2_	*Z* = 2
*M**_r_* = 613.96	*F*(000) = 636
Triclinic, *P*1	*D*_x_ = 1.344 Mg m^−^^3^
Hall symbol: -P 1	Mo *K*α radiation, λ = 0.71073 Å
*a* = 9.974 (6) Å	Cell parameters from 1683 reflections
*b* = 12.546 (7) Å	θ = 2.5–27.8°
*c* = 12.798 (8) Å	µ = 0.86 mm^−^^1^
α = 96.631 (11)°	*T* = 296 K
β = 97.016 (12)°	Block, colourless
γ = 105.060 (12)°	0.16 × 0.14 × 0.12 mm
*V* = 1516.7 (16) Å^3^	

### Data collection

**Table:**

Bruker APEXII CCD area-detector diffractometer	5295 independent reflections
Radiation source: fine-focus sealed tube	2785 reflections with *I* > 2σ(*I*)
graphite	*R*_int_ = 0.046
φ– and ω–scans	θ_max_ = 25.0°, θ_min_ = 1.6°
Absorption correction: multi-scan (*SADABS*; Bruker, 2005)	*h* = −10→11
*T*_min_ = 0.875, *T*_max_ = 0.904	*k* = −14→13
8123 measured reflections	*l* = −15→15

### Refinement

**Table:**

Refinement on *F*^2^	Primary atom site location: structure-invariant direct methods
Least-squares matrix: full	Secondary atom site location: difference Fourier map
*R*[*F*^2^ > 2σ(*F*^2^)] = 0.065	Hydrogen site location: inferred from neighbouring sites
*wR*(*F*^2^) = 0.136	H-atom parameters constrained
*S* = 1.04	*w* = 1/[σ^2^(*F*_o_^2^) + (0.0455*P*)^2^] where *P* = (*F*_o_^2^ + 2*F*_c_^2^)/3
5295 reflections	(Δ/σ)_max_ < 0.001
370 parameters	Δρ_max_ = 0.64 e Å^−^^3^
14 restraints	Δρ_min_ = −0.64 e Å^−^^3^

### Special details

**Table:**

Geometry. All s.u.'s (except the s.u. in the dihedral angle between two l.s. planes) are estimated using the full covariance matrix. The cell s.u.'s are taken into account individually in the estimation of s.u.'s in distances, angles and torsion angles; correlations between s.u.'s in cell parameters are only used when they are defined by crystal symmetry. An approximate (isotropic) treatment of cell s.u.'s is used for estimating s.u.'s involving l.s. planes.
Refinement. Refinement of F^2^ against ALL reflections. The weighted R-factor wR and goodness of fit S are based on F^2^, conventional R-factors R are based on F, with F set to zero for negative F^2^. The threshold expression of F^2^ > 2σ(F^2^) is used only for calculating R-factors(gt) etc. and is not relevant to the choice of reflections for refinement. R-factors based on F^2^ are statistically about twice as large as those based on F, and R-factors based on ALL data will be even larger.

### Fractional atomic coordinates and isotropic or equivalent isotropic displacement parameters (Å^2^)

**Table:**

	*x*	*y*	*z*	*U*_iso_*/*U*_eq_	
Zn1	0.41555 (6)	0.22239 (5)	0.15235 (4)	0.0561 (2)	
N1	0.2722 (4)	0.0734 (3)	0.0835 (3)	0.0509 (10)	
N2	0.4792 (4)	0.2028 (3)	0.0057 (3)	0.0461 (10)	
O1	0.6236 (4)	0.2621 (4)	0.2360 (3)	0.1060 (12)	
O2	0.4751 (4)	0.1472 (3)	0.2994 (3)	0.0945 (13)	
O3	0.3506 (4)	0.3454 (3)	0.2070 (3)	0.0708 (10)	
O4	0.2026 (4)	0.3185 (3)	0.0566 (2)	0.0672 (10)	
O5	0.1962 (4)	0.1688 (3)	0.8042 (3)	0.0760 (11)	
O6	0.2806 (4)	0.3497 (3)	0.8688 (3)	0.0717 (10)	
H6	0.2520	0.3287	0.9225	0.108*	
C1	0.5999 (8)	0.1987 (7)	0.3044 (5)	0.1025 (12)	
C2	0.7122 (5)	0.1859 (4)	0.3773 (3)	0.0997 (13)	
C3	0.8523 (6)	0.2392 (4)	0.3750 (4)	0.128 (3)	
H3	0.8755	0.2882	0.3265	0.153*	
C4	0.9579 (4)	0.2192 (5)	0.4450 (5)	0.164 (4)	
H4	1.0517	0.2549	0.4434	0.196*	
C5	0.9232 (6)	0.1460 (5)	0.5174 (4)	0.172 (5)	
H5	0.9938	0.1326	0.5642	0.206*	
C6	0.7831 (7)	0.0926 (4)	0.5197 (4)	0.153 (4)	
H6A	0.7599	0.0436	0.5681	0.183*	
C7	0.6775 (5)	0.1126 (4)	0.4497 (4)	0.111 (3)	
H7	0.5837	0.0769	0.4513	0.134*	
C8	0.2506 (6)	0.3676 (4)	0.1480 (4)	0.0535 (13)	
C9	0.1893 (5)	0.4536 (4)	0.1966 (4)	0.0537 (13)	
C10	0.2524 (7)	0.5206 (5)	0.2920 (4)	0.0894 (19)	
H10	0.3346	0.5114	0.3277	0.107*	
C11	0.1952 (9)	0.6015 (6)	0.3357 (5)	0.116 (2)	
H11	0.2383	0.6463	0.4003	0.139*	
C12	0.0740 (9)	0.6148 (6)	0.2822 (7)	0.125 (3)	
H12	0.0324	0.6668	0.3120	0.150*	
C13	0.0151 (7)	0.5522 (6)	0.1864 (7)	0.117 (3)	
H13	−0.0654	0.5631	0.1496	0.140*	
C14	0.0726 (6)	0.4730 (4)	0.1430 (5)	0.0780 (17)	
H14	0.0320	0.4318	0.0763	0.094*	
C15	0.2525 (5)	0.2627 (5)	0.7920 (4)	0.0529 (13)	
C16	0.2973 (5)	0.2925 (5)	0.6902 (4)	0.0538 (13)	
C17	0.3171 (6)	0.3999 (5)	0.6671 (4)	0.0758 (16)	
H17	0.3070	0.4564	0.7167	0.091*	
C18	0.3528 (7)	0.4224 (6)	0.5677 (5)	0.097 (2)	
H18	0.3660	0.4941	0.5508	0.116*	
C19	0.3684 (7)	0.3383 (7)	0.4953 (5)	0.098 (2)	
H19	0.3910	0.3533	0.4291	0.118*	
C20	0.3510 (6)	0.2331 (6)	0.5199 (5)	0.0866 (19)	
H20	0.3653	0.1771	0.4720	0.104*	
C21	0.3122 (5)	0.2107 (5)	0.6159 (4)	0.0689 (15)	
H21	0.2955	0.1380	0.6308	0.083*	
C22	0.1317 (6)	0.0689 (5)	0.2271 (4)	0.0847 (18)	
H22A	0.2067	0.0747	0.2840	0.127*	
H22B	0.0452	0.0241	0.2431	0.127*	
H22C	0.1232	0.1421	0.2192	0.127*	
C23	0.1631 (6)	0.0156 (5)	0.1256 (4)	0.0633 (15)	
C24	0.0820 (6)	−0.0880 (5)	0.0732 (6)	0.0798 (18)	
H24	0.0049	−0.1267	0.1009	0.096*	
C25	0.1145 (7)	−0.1335 (5)	−0.0189 (6)	0.087 (2)	
H25	0.0622	−0.2045	−0.0524	0.105*	
C26	0.2243 (6)	−0.0747 (4)	−0.0621 (4)	0.0719 (16)	
H26	0.2461	−0.1040	−0.1259	0.086*	
C27	0.3021 (5)	0.0296 (4)	−0.0087 (4)	0.0482 (12)	
C28	0.4195 (5)	0.1021 (4)	−0.0519 (4)	0.0471 (12)	
C29	0.4627 (6)	0.0693 (5)	−0.1451 (4)	0.0648 (15)	
H29	0.4218	−0.0020	−0.1832	0.078*	
C30	0.5682 (7)	0.1441 (6)	−0.1813 (4)	0.0785 (17)	
H30	0.5997	0.1236	−0.2440	0.094*	
C31	0.6250 (6)	0.2471 (5)	−0.1248 (5)	0.0753 (16)	
H31	0.6951	0.2982	−0.1492	0.090*	
C32	0.5798 (6)	0.2774 (4)	−0.0311 (4)	0.0601 (14)	
C33	0.6386 (6)	0.3902 (4)	0.0338 (5)	0.095 (2)	
H33A	0.5630	0.4200	0.0507	0.142*	
H33B	0.6962	0.4388	−0.0060	0.142*	
H33C	0.6944	0.3846	0.0985	0.142*	

### Atomic displacement parameters (Å^2^)

**Table:**

	*U*^11^	*U*^22^	*U*^33^	*U*^12^	*U*^13^	*U*^23^
Zn1	0.0556 (4)	0.0578 (4)	0.0511 (4)	0.0185 (3)	−0.0002 (3)	−0.0035 (3)
N1	0.040 (2)	0.057 (3)	0.053 (3)	0.013 (2)	−0.002 (2)	0.013 (2)
N2	0.038 (2)	0.046 (3)	0.054 (2)	0.016 (2)	0.005 (2)	0.002 (2)
O1	0.0884 (18)	0.162 (3)	0.069 (2)	0.063 (2)	−0.0244 (18)	−0.003 (2)
O2	0.093 (3)	0.124 (3)	0.066 (2)	0.061 (3)	−0.034 (2)	−0.0054 (18)
O3	0.081 (3)	0.074 (2)	0.056 (2)	0.037 (2)	−0.008 (2)	−0.0079 (18)
O4	0.078 (3)	0.075 (2)	0.044 (2)	0.0216 (19)	0.0045 (19)	−0.0047 (18)
O5	0.092 (3)	0.073 (3)	0.065 (2)	0.021 (2)	0.019 (2)	0.014 (2)
O6	0.100 (3)	0.068 (2)	0.051 (2)	0.030 (2)	0.016 (2)	0.0073 (19)
C1	0.086 (2)	0.158 (3)	0.066 (2)	0.065 (2)	−0.0219 (19)	−0.006 (2)
C2	0.084 (2)	0.155 (4)	0.064 (2)	0.066 (2)	−0.021 (2)	−0.007 (2)
C3	0.075 (5)	0.201 (8)	0.097 (5)	0.050 (5)	0.003 (4)	−0.027 (5)
C4	0.096 (6)	0.256 (12)	0.121 (7)	0.078 (7)	−0.032 (6)	−0.055 (7)
C5	0.135 (9)	0.205 (11)	0.155 (9)	0.101 (8)	−0.086 (7)	−0.069 (8)
C6	0.211 (10)	0.094 (6)	0.134 (7)	0.068 (6)	−0.083 (7)	−0.003 (5)
C7	0.117 (6)	0.095 (5)	0.100 (5)	0.043 (4)	−0.057 (5)	−0.030 (4)
C8	0.068 (4)	0.049 (3)	0.042 (3)	0.014 (3)	0.014 (3)	0.003 (3)
C9	0.059 (3)	0.053 (3)	0.047 (3)	0.019 (3)	0.006 (3)	−0.002 (3)
C10	0.120 (5)	0.095 (5)	0.063 (4)	0.058 (4)	0.004 (4)	−0.005 (3)
C11	0.160 (8)	0.120 (6)	0.075 (5)	0.071 (6)	0.012 (5)	−0.024 (4)
C12	0.114 (7)	0.120 (6)	0.147 (7)	0.064 (5)	0.025 (6)	−0.030 (6)
C13	0.071 (5)	0.101 (6)	0.166 (8)	0.039 (4)	−0.012 (5)	−0.033 (5)
C14	0.060 (4)	0.068 (4)	0.096 (4)	0.021 (3)	−0.003 (4)	−0.016 (3)
C15	0.048 (3)	0.059 (4)	0.055 (3)	0.022 (3)	0.005 (3)	0.011 (3)
C16	0.049 (3)	0.066 (4)	0.045 (3)	0.016 (3)	0.002 (3)	0.009 (3)
C17	0.075 (4)	0.082 (5)	0.065 (4)	0.012 (3)	0.011 (3)	0.013 (3)
C18	0.103 (5)	0.101 (5)	0.074 (4)	−0.001 (4)	0.018 (4)	0.028 (4)
C19	0.090 (5)	0.141 (7)	0.055 (4)	0.014 (5)	0.017 (4)	0.018 (5)
C20	0.084 (5)	0.117 (6)	0.060 (4)	0.031 (4)	0.015 (3)	0.002 (4)
C21	0.072 (4)	0.082 (4)	0.054 (3)	0.026 (3)	0.009 (3)	0.006 (3)
C22	0.063 (4)	0.116 (5)	0.078 (4)	0.021 (4)	0.014 (3)	0.026 (4)
C23	0.052 (4)	0.069 (4)	0.069 (4)	0.018 (3)	−0.006 (3)	0.025 (3)
C24	0.058 (4)	0.065 (5)	0.110 (5)	0.003 (3)	0.001 (4)	0.034 (4)
C25	0.075 (5)	0.056 (4)	0.114 (6)	0.004 (4)	−0.017 (4)	0.008 (4)
C26	0.069 (4)	0.054 (4)	0.079 (4)	0.011 (3)	−0.012 (3)	−0.004 (3)
C27	0.046 (3)	0.043 (3)	0.051 (3)	0.015 (3)	−0.012 (3)	0.002 (3)
C28	0.047 (3)	0.046 (3)	0.051 (3)	0.025 (3)	−0.003 (3)	0.000 (3)
C29	0.076 (4)	0.061 (4)	0.060 (4)	0.032 (3)	0.005 (3)	−0.008 (3)
C30	0.096 (5)	0.104 (5)	0.055 (4)	0.053 (4)	0.030 (4)	0.016 (4)
C31	0.073 (4)	0.086 (5)	0.074 (4)	0.030 (4)	0.019 (3)	0.017 (4)
C32	0.060 (4)	0.056 (4)	0.064 (4)	0.019 (3)	0.005 (3)	0.009 (3)
C33	0.099 (5)	0.066 (4)	0.099 (4)	−0.012 (3)	0.023 (4)	0.005 (4)

### Geometric parameters (Å, °)

**Table:**

Zn1—O3	1.917 (3)	C13—H13	0.9300
Zn1—N1	2.059 (4)	C14—H14	0.9300
Zn1—N2	2.062 (4)	C15—C16	1.484 (7)
Zn1—O1	2.120 (4)	C16—C21	1.368 (6)
Zn1—O2	2.283 (4)	C16—C17	1.381 (7)
N1—C27	1.344 (6)	C17—C18	1.402 (7)
N1—C23	1.350 (6)	C17—H17	0.9300
N2—C28	1.335 (5)	C18—C19	1.375 (8)
N2—C32	1.353 (6)	C18—H18	0.9300
O1—C1	1.252 (8)	C19—C20	1.365 (8)
O2—C1	1.235 (8)	C19—H19	0.9300
O3—C8	1.285 (5)	C20—C21	1.369 (7)
O4—C8	1.233 (5)	C20—H20	0.9300
O5—C15	1.204 (5)	C21—H21	0.9300
O6—C15	1.327 (5)	C22—C23	1.496 (7)
O6—H6	0.8200	C22—H22A	0.9600
C1—C2	1.423 (7)	C22—H22B	0.9600
C2—C3	1.3900	C22—H22C	0.9600
C2—C7	1.3900	C23—C24	1.384 (7)
C3—C4	1.3900	C24—C25	1.362 (8)
C3—H3	0.9300	C24—H24	0.9300
C4—C5	1.3900	C25—C26	1.368 (8)
C4—H4	0.9300	C25—H25	0.9300
C5—C6	1.3900	C26—C27	1.384 (6)
C5—H5	0.9300	C26—H26	0.9300
C6—C7	1.3900	C27—C28	1.492 (6)
C6—H6A	0.9300	C28—C29	1.372 (6)
C7—H7	0.9300	C29—C30	1.382 (7)
C8—C9	1.485 (7)	C29—H29	0.9300
C9—C14	1.369 (6)	C30—C31	1.349 (7)
C9—C10	1.377 (6)	C30—H30	0.9300
C10—C11	1.384 (8)	C31—C32	1.379 (7)
C10—H10	0.9300	C31—H31	0.9300
C11—C12	1.374 (9)	C32—C33	1.488 (7)
C11—H11	0.9300	C33—H33A	0.9600
C12—C13	1.352 (8)	C33—H33B	0.9600
C12—H12	0.9300	C33—H33C	0.9600
C13—C14	1.369 (8)		
			
O3—Zn1—N1	119.74 (16)	O5—C15—C16	123.5 (5)
O3—Zn1—N2	124.65 (15)	O6—C15—C16	113.6 (5)
N1—Zn1—N2	80.07 (17)	C21—C16—C17	119.5 (5)
O3—Zn1—O1	102.78 (16)	C21—C16—C15	119.1 (5)
N1—Zn1—O1	132.61 (16)	C17—C16—C15	121.3 (5)
N2—Zn1—O1	93.04 (16)	C16—C17—C18	119.0 (6)
O3—Zn1—O2	104.33 (15)	C16—C17—H17	120.5
N1—Zn1—O2	91.25 (15)	C18—C17—H17	120.5
N2—Zn1—O2	127.89 (14)	C19—C18—C17	120.0 (6)
O1—Zn1—O2	56.64 (17)	C19—C18—H18	120.0
C27—N1—C23	119.8 (4)	C17—C18—H18	120.0
C27—N1—Zn1	113.4 (3)	C20—C19—C18	120.4 (6)
C23—N1—Zn1	126.6 (4)	C20—C19—H19	119.8
C28—N2—C32	119.6 (4)	C18—C19—H19	119.8
C28—N2—Zn1	113.8 (3)	C19—C20—C21	119.4 (6)
C32—N2—Zn1	126.4 (3)	C19—C20—H20	120.3
C1—O1—Zn1	98.0 (4)	C21—C20—H20	120.3
C1—O2—Zn1	90.6 (4)	C20—C21—C16	121.6 (6)
C8—O3—Zn1	117.4 (3)	C20—C21—H21	119.2
C15—O6—H6	109.5	C16—C21—H21	119.2
O2—C1—O1	114.7 (6)	C23—C22—H22A	109.5
O2—C1—C2	124.5 (7)	C23—C22—H22B	109.5
O1—C1—C2	120.8 (7)	H22A—C22—H22B	109.5
C3—C2—C7	120.0	C23—C22—H22C	109.5
C3—C2—C1	122.6 (5)	H22A—C22—H22C	109.5
C7—C2—C1	117.3 (5)	H22B—C22—H22C	109.5
C4—C3—C2	120.0	N1—C23—C24	119.8 (5)
C4—C3—H3	120.0	N1—C23—C22	117.9 (5)
C2—C3—H3	120.0	C24—C23—C22	122.2 (6)
C3—C4—C5	120.0	C25—C24—C23	120.2 (6)
C3—C4—H4	120.0	C25—C24—H24	119.9
C5—C4—H4	120.0	C23—C24—H24	119.9
C4—C5—C6	120.0	C24—C25—C26	120.0 (6)
C4—C5—H5	120.0	C24—C25—H25	120.0
C6—C5—H5	120.0	C26—C25—H25	120.0
C7—C6—C5	120.0	C25—C26—C27	118.4 (6)
C7—C6—H6A	120.0	C25—C26—H26	120.8
C5—C6—H6A	120.0	C27—C26—H26	120.8
C6—C7—C2	120.0	N1—C27—C26	121.7 (5)
C6—C7—H7	120.0	N1—C27—C28	115.9 (4)
C2—C7—H7	120.0	C26—C27—C28	122.4 (5)
O4—C8—O3	123.0 (5)	N2—C28—C29	121.7 (5)
O4—C8—C9	120.3 (5)	N2—C28—C27	115.4 (4)
O3—C8—C9	116.7 (4)	C29—C28—C27	122.9 (5)
C14—C9—C10	118.3 (5)	C28—C29—C30	118.8 (5)
C14—C9—C8	120.0 (5)	C28—C29—H29	120.6
C10—C9—C8	121.6 (5)	C30—C29—H29	120.6
C9—C10—C11	121.0 (6)	C31—C30—C29	119.3 (5)
C9—C10—H10	119.5	C31—C30—H30	120.4
C11—C10—H10	119.5	C29—C30—H30	120.4
C12—C11—C10	119.1 (6)	C30—C31—C32	120.5 (6)
C12—C11—H11	120.5	C30—C31—H31	119.7
C10—C11—H11	120.5	C32—C31—H31	119.7
C13—C12—C11	120.0 (7)	N2—C32—C31	120.0 (5)
C13—C12—H12	120.0	N2—C32—C33	117.5 (5)
C11—C12—H12	120.0	C31—C32—C33	122.5 (5)
C12—C13—C14	120.8 (7)	C32—C33—H33A	109.5
C12—C13—H13	119.6	C32—C33—H33B	109.5
C14—C13—H13	119.6	H33A—C33—H33B	109.5
C9—C14—C13	120.8 (6)	C32—C33—H33C	109.5
C9—C14—H14	119.6	H33A—C33—H33C	109.5
C13—C14—H14	119.6	H33B—C33—H33C	109.5
O5—C15—O6	122.9 (5)		
			
O3—Zn1—N1—C27	134.4 (3)	C8—C9—C10—C11	179.1 (6)
N2—Zn1—N1—C27	10.0 (3)	C9—C10—C11—C12	−0.2 (11)
O1—Zn1—N1—C27	−74.9 (4)	C10—C11—C12—C13	−2.5 (13)
O2—Zn1—N1—C27	−118.3 (3)	C11—C12—C13—C14	1.9 (13)
O3—Zn1—N1—C23	−50.8 (4)	C10—C9—C14—C13	−4.0 (9)
N2—Zn1—N1—C23	−175.2 (4)	C8—C9—C14—C13	−179.8 (6)
O1—Zn1—N1—C23	99.9 (4)	C12—C13—C14—C9	1.4 (11)
O2—Zn1—N1—C23	56.5 (4)	O5—C15—C16—C21	−18.5 (7)
O3—Zn1—N2—C28	−130.2 (3)	O6—C15—C16—C21	161.4 (4)
N1—Zn1—N2—C28	−10.8 (3)	O5—C15—C16—C17	158.1 (5)
O1—Zn1—N2—C28	122.0 (3)	O6—C15—C16—C17	−22.0 (6)
O2—Zn1—N2—C28	73.0 (3)	C21—C16—C17—C18	−0.3 (8)
O3—Zn1—N2—C32	55.2 (4)	C15—C16—C17—C18	−176.9 (5)
N1—Zn1—N2—C32	174.6 (4)	C16—C17—C18—C19	−0.4 (9)
O1—Zn1—N2—C32	−52.6 (4)	C17—C18—C19—C20	−0.8 (10)
O2—Zn1—N2—C32	−101.6 (4)	C18—C19—C20—C21	2.6 (10)
O3—Zn1—O1—C1	100.7 (4)	C19—C20—C21—C16	−3.4 (9)
N1—Zn1—O1—C1	−53.5 (5)	C17—C16—C21—C20	2.2 (8)
N2—Zn1—O1—C1	−132.8 (4)	C15—C16—C21—C20	178.9 (5)
O2—Zn1—O1—C1	1.8 (4)	C27—N1—C23—C24	−0.5 (7)
O3—Zn1—O2—C1	−97.8 (4)	Zn1—N1—C23—C24	−175.0 (4)
N1—Zn1—O2—C1	141.0 (4)	C27—N1—C23—C22	−178.6 (4)
N2—Zn1—O2—C1	62.6 (4)	Zn1—N1—C23—C22	6.9 (6)
O1—Zn1—O2—C1	−1.8 (4)	N1—C23—C24—C25	2.1 (8)
N1—Zn1—O3—C8	−45.9 (4)	C22—C23—C24—C25	−179.9 (5)
N2—Zn1—O3—C8	52.9 (4)	C23—C24—C25—C26	−2.7 (9)
O1—Zn1—O3—C8	155.8 (3)	C24—C25—C26—C27	1.6 (8)
O2—Zn1—O3—C8	−145.8 (3)	C23—N1—C27—C26	−0.6 (6)
Zn1—O2—C1—O1	2.8 (6)	Zn1—N1—C27—C26	174.6 (3)
Zn1—O2—C1—C2	−174.3 (6)	C23—N1—C27—C28	176.8 (4)
Zn1—O1—C1—O2	−3.0 (7)	Zn1—N1—C27—C28	−7.9 (5)
Zn1—O1—C1—C2	174.2 (5)	C25—C26—C27—N1	0.1 (7)
O2—C1—C2—C3	175.0 (5)	C25—C26—C27—C28	−177.2 (5)
O1—C1—C2—C3	−1.9 (8)	C32—N2—C28—C29	3.7 (6)
O2—C1—C2—C7	−2.0 (8)	Zn1—N2—C28—C29	−171.4 (3)
O1—C1—C2—C7	−178.9 (5)	C32—N2—C28—C27	−175.3 (4)
C7—C2—C3—C4	0.0	Zn1—N2—C28—C27	9.7 (4)
C1—C2—C3—C4	−176.9 (5)	N1—C27—C28—N2	−1.2 (5)
C2—C3—C4—C5	0.0	C26—C27—C28—N2	176.3 (4)
C3—C4—C5—C6	0.0	N1—C27—C28—C29	179.9 (4)
C4—C5—C6—C7	0.0	C26—C27—C28—C29	−2.7 (7)
C5—C6—C7—C2	0.0	N2—C28—C29—C30	−1.8 (7)
C3—C2—C7—C6	0.0	C27—C28—C29—C30	177.1 (4)
C1—C2—C7—C6	177.1 (4)	C28—C29—C30—C31	−0.5 (8)
Zn1—O3—C8—O4	−5.0 (6)	C29—C30—C31—C32	0.8 (8)
Zn1—O3—C8—C9	172.6 (3)	C28—N2—C32—C31	−3.3 (7)
O4—C8—C9—C14	4.8 (7)	Zn1—N2—C32—C31	171.1 (3)
O3—C8—C9—C14	−172.9 (5)	C28—N2—C32—C33	177.4 (4)
O4—C8—C9—C10	−170.8 (5)	Zn1—N2—C32—C33	−8.2 (6)
O3—C8—C9—C10	11.5 (7)	C30—C31—C32—N2	1.0 (8)
C14—C9—C10—C11	3.4 (9)	C30—C31—C32—C33	−179.7 (5)

### Hydrogen-bond geometry (Å, °)

**Table:**

*D*—H···*A*	*D*—H	H···*A*	*D*···*A*	*D*—H···*A*
C24—H24···O5^i^	0.93	2.44	3.340 (8)	162
C33—H33C···O1	0.96	2.52	3.199 (8)	128
C22—H22A···O2	0.96	2.57	3.289 (7)	132
O6—H6···O4^ii^	0.82	1.85	2.655 (5)	166
C31—H31···Cg1^iii^	0.93	2.79	3.708 (7)	170

Symmetry codes: (i) −*x*, −*y*, −*z*+1; (ii) *x*, *y*, *z*+1; (iii) −*x*+1, −*y*+1, −*z*.

## References

[bb1] Alizadeh, R., Kalateh, K., Ebadi, A., Ahmadi, R. & Amani, V. (2009). *Acta Cryst.* E**65**, m1250. 10.1107/S1600536809038215 PMC2970226 21577766

[bb2] Bruker (2005). *APEX2*, *SAINT* and *SADABS* Bruker AXS Inc., Madison, Wisconsin, USA.

[bb3] Cui, G. H., Li, J. R., Gao, D. & Ng, S. W. (2005). *Acta Cryst.* E**61**, m72–m73.

[bb4] Hökelek, T., Dal, H., Tercan, B., Aybirdi, Ö. & Necefoğlu, H. (2009*a*). *Acta Cryst. *E**65**, m1037–m1038. 10.1107/S1600536809027093 PMC2970058 21577401

[bb5] Hökelek, T., Dal, H., Tercan, B., Aybirdi, Ö. & Necefoğlu, H. (2009*b*). *Acta Cryst.* E**65**, m1051–m1052. 10.1107/S1600536809030980 PMC2970090 21577412

[bb6] Klausmeyer, K. K., Hung-Low, F. & Renz, A. (2007). *Acta Cryst.* E**63**, m2181.

[bb7] Phatchimkun, J., Kongsaeree, P., Suchaichit, N. & Chaichit, N. (2009). *Acta Cryst.* E**65**, m1020–m1021. 10.1107/S1600536809029407 PMC2970126 21577389

[bb8] Sheldrick, G. M. (2008). *Acta Cryst.* A**64**, 112–122. 10.1107/S0108767307043930 18156677

[bb9] Zhang, B.-Y., Nie, J.-J. & Xu, D.-J. (2009). *Acta Cryst.* E**65**, m880. 10.1107/S1600536809025525 PMC2977467 21583344

